# Identifying barriers and finding solutions to implement best practices for cancer surgery at Maputo Central Hospital, Mozambique

**DOI:** 10.3332/ecancer.2018.878

**Published:** 2018-10-23

**Authors:** Atílio Morais, Matchecane Cossa, Adriano Tivane, Jotamo Come, Volodimir Venetsky, Fernando Torres, Victor Pacheco, Miguel Reyes, Germano Pires, Mariana Peyroteo, Satish Tulsidas, Ellen Baker, Moshin Sidat, Maria do Rosário O Martins, Lúcio Lara Santos

**Affiliations:** 1 Thoracic Surgery, Surgical Department, Maputo Central Hospital, Av Agostinho Neto n° 164, Maputo 1164, Mozambique; 2 Breast Surgery, Surgical Department, Maputo Central Hospital, Av Agostinho Neto n° 164, Maputo 1164, Mozambique; 3 Colorectal Surgery, Surgical Department, Maputo Central Hospital, Av Agostinho Neto n° 164, Maputo 1164, Mozambique; 4 Heath National Institute, Av Eduardo Mondlane, Maputo 264, Mozambique; 5 Surgical Oncology Department, Portuguese Institute of Oncology, Dr António Bernardino de Almeida Street, Porto 4200-072, Portugal; 6 Medical Oncology Department, Maputo Central Hospital, Av Agostinho Neto n° 164, Maputo 1164, Mozambique; 7 UT MD Anderson Cancer Center, 1515 Holcombe Blvd, Houston, TX 77030, USA; 8 Department of Community Health, University Eduardo Mondlane, Av Julius Nyerere, Maputo 257, Mozambique; 9 Global Health and Tropical Medicine, Institute of Hygiene and Tropical Medicine, Universidade Nova de Lisboa, Junqueira Street 100, Lisboa 1349-008 Portugal; 10 Experimental Pathology and Therapeutics Research Group, Surgical Oncology Department, Portuguese Institute of Oncology, Dr António Bernardino de Almeida Street, Porto 4200-072, Portugal; 11 ONCOCIR—Education and Care in Oncology—Lusophone Africa, Quires Street 168, Moreira da Maia 4470-643, Portugal

**Keywords:** training, surgical oncology, curriculum development, Mozambique

## Abstract

**Purpose:**

The aim of this study was to assess the surgical resources and surgical oncology team skills at the Surgical Department of Maputo Central Hospital (MCH) in Mozambique in order to define an educational program to support surgical oncology practice.

**Methods:**

From January 2017 to December 2017, a general evaluation of the resources of MCH was carried out, as well as its offerings in oncological care in different services. Data were obtained by reviewing documents, visiting surgical services and interviewing key-informants and others informally. In addition, a group of seven surgeons of the Surgical Department of MCH answered a questionnaire about the quality of the cancer units (The Cancer Units Assessment Checklist for low- or middle-income African countries). Subsequently, surgical, anaesthesiology and intensive care facilities were evaluated according to the Portuguese-speaking African Countries Assessment of Surgical Oncology Capacity Survey (PSAC-Surgery). All the data were triangulated in order to identify gaps, develop an action plan and define an educational program.

**Results:**

Breast, oesophagus and colorectal cancers were the most commonly treated neoplasms in MCH. A range of technical and resource needs as well as the gaps in knowledge and skills were identified. All surgeons recognised the need to create a training program in oncology at the undergraduate level, specific training for residents and continuing oncological education for general surgeons to improve the practice of surgical oncology. It was evident that all these interventions needed to be formalised, appropriately certified and count for professional career progression. Based on the local epidemiological data and on these study findings, oncology education programs were developed for surgeons.

**Conclusions:**

The findings of this study contributed to the development of an educational program in surgical oncology, considered essential to the training of surgeons at MCH. The cancer educational programs and the mobilisation of adequate resources will ensure the provision of adequate surgical oncology treatments for MCH. The training requirements should be tailored to suit the local needs based on the most prevalent malignancies diagnosed in the region. In our view, this methodology may apply to other countries with similar realities in the formation of surgical oncologists.

## Introduction

Cancer is a major public health problem in Sub-Saharan Africa because of population aging, as well as increased prevalence of key risk factors [1]. The resources available for cancer control are less than adequate in Africa; therefore, standardised cancer treatment for low- and middle-income African countries (LMIACs) is still a major concern and surgery plays an important role in the diagnosis, staging and treatment of cancer [2, 3]. In most LMIACs, as Mozambique, surgery remains the only locoregional treatment and represents the best hope for the cure, thus a careful evaluation of surgical team skills is critical to improving cancer management [4]. In addition, the quantity, quality and functionality of equipment and supplies, availability of running water and electricity, access to safe blood transfusion services, chemotherapy and radiation, the presence of postoperative facilities as well as the number, type and qualification of healthcare personnel should be included in the assessment of cancer surgery quality [4]. Noncommunicable diseases, including cancer, have been considered a major public health problem by the Ministry of Health of Mozambique since 2008 [5].

According to Lorenzoni *et al* [6], in males, the most common cancers are prostate, Kaposi sarcoma (KS), liver and oesophagus. In females, the most frequent cancers are cervix, breast, KS and oesophagus.

The report on human resources needed for cancer control in low- and middle-income countries, performed by the National Cancer Institute suggested that surgical oncologists, medical oncologists and radiation oncologists are an unmet need in Mozambique. In addition, support staffs such as onco-pharmacists, pharmacy technicians, oncology nurses and palliative care specialists are also needed. Recently, an article evaluating the cancer plans for Mozambique the authors revealed that there are only seven oncologists in the country and no oncological surgeons [7, 8]. Thus, an educational cancer program is crucial to existing surgical teams and medical students. According to Snyder *et al* [9], 14% of the causes of death in the Maputo Central Hospital (MCH) surgery department are oncological diseases and neoplasms accounted for 9% of all surgical discharge diagnoses [9]. The cancer registry of the MCH in 2015–16 pointed out that the malignant tumours most often operated by general and thoracic surgery are breast, oesophageal and colorectal cancer [10].

In order to develop and implement a Cancer Education Program (CEP) and introduce best practices for cancer surgery at MCH, Mozambique, we assessed current surgical resources and surgical oncology skills.

## Methods

This cross-sectional study was conducted between January 2017 and December 2017 in MCH, a tertiary level hospital in Mozambique. An evaluation of the general capacity of MCH and its offer in oncological care in the different services was done through documental analysis, visits to the services and informal interviews to the providers of oncological care in those services. In addition, a questionnaire was administered to the seven general surgeons of the surgical Department of MCH, who have the main role in the surgical treatment of the most frequent types of cancer, namely, breast (three surgeons), colorectal (two surgeons) and oesophageal cancer (two surgeons).

The questionnaire used was the cancer units assessment checklist for low- or middle-income African countries [11]. Subsequently, surgical, anaesthesiology and intensive-care facilities were evaluated according to the Portuguese-speaking African Countries Assessment for Surgical Oncology Capacity Survey (PSAC-Surgery) in order to identify gaps.

This second instrument assesses the capacity of the hospital to perform surgical oncological procedures; infrastructures available; specific resources to perform breast, oesophageal and colorectal cancer surgery; workforce available, and the number of surgical oncology procedures per year.

The institutions that train health professionals in Mozambique were also evaluated in order to understand the formative capacity and the potential integration of a CEP in their curriculum.

After the visits, interviews, documental analysis and questionnaires assessment, the main needs in general oncology and surgical oncology were identified, in particular, for residents and fellows in general surgery. Moreover, a set of recommendations for the training of general surgeons at MCH Surgical Department were also suggested.

Questionnaires were collected on paper and the data were entered into an electronic database. Descriptive statistics were performed. This study was approved for the Mozambican National Bioethical Committee.

## Results

MCH, a tertiary unit level (1500 beds), is the referral centre for all of the complex surgical care in the country. MCH provides a complete emergency service with advanced diagnostic capacities, inpatient wards for complex medical and surgical care (325 beds in the surgical unit), three fully equipped operating rooms, a fully equipped delivery room, three recovery rooms, an intensive-care unit, two high dependency care units and rehabilitation therapy facilities. The centre is also equipped with respirators, oxygen supply devices, intravenous fluids, blood products, basic microbiology equipment, the main pharmaceuticals (anaesthetics, analgesics, antibiotics) and the main surgical materials (drapes, gowns, dressings, gloves) as well as other consumables (disposable equipment and devices).

Human resources for health at MCH include: nurses—700, operating room nurses—30, anaesthetists nurses—15, anaesthesiologists—20, general physicians—125, obstetricians/gynaecologists—20, general surgeons—10, orthopaedic surgeons—7, pharmacy assistants—40, pharmacists—10, radiology technicians—12, radiologists—3, physiotherapists—2, neurosurgeons—5, thoracic surgeons—3, reconstructive surgeons—3, urologists—4, medical oncologists—3, radio-oncologist—1, medical physics—3, gastroenterologists—4 and pathologists—10.

The first questionnaire was fulfilled by seven surgeons. Cancer diagnosis pitfalls were related with fragile imaging resources and low capacity to perform biopsies guides per image. The specific needs to properly diagnose and treat the most common tumours are summarised in Table 1.

For treatment capacities, the following needs were identified: existence of a safe oncology pharmacy with access to essential oncology drugs according to the WHO list; the improvement of the day hospital facilities; to start the activity of the radiotherapy unit; to endow the surgical services with the missing resources and to produce guidelines to ensure good practice in oncology. Other needs include pre-rehabilitation programs for frail patients, rehabilitation and palliative care. At present, there are no surgeons with formal training and certification in surgical oncology.

All surgeons recognised the need to create a training program in oncology at the undergraduate level. Table 2 shows the training capacity of health professionals in Mozambique where this training should take place. Moreover, a specific training for residents and fellows (wherever possible with practical training in high workload centres) and continuing oncological education for general surgeons to improve the practice of surgical oncology was also recommended (Table 3). Surgeons also underlined that their participation in multidisciplinary decision-making meetings (locally known as the tumour Commission) is infrequent; they also stressed the need to improve the oncological knowledge about the most frequent tumours, scientific research and how to strength external collaborations.

Since the most frequently diagnosed tumours surgically treated at MCH are breast, oesophageal and colorectal cancer and based on the gaps mentioned by the seven surgeons, a Global Curriculum in Surgical Oncology was elaborated and will be tested at the MCH (Table 4) [12].

## Discussion

MCH, the referral centre for all complex surgical care in Mozambique, provides primary surgical care for its local population and is also a teaching hospital. In MCH, the top three solid tumours treated by general and thoracic surgery are breast, oesophageal and colorectal cancer.

This study was particularly helpful to define the global needs of cancer education at the national level and, in particular, at MCH. The results from the first questionnaires revealed a shortage of both technical and material resources and skills/formative deficits. In addition, direct observation through the visit to operating rooms, intensive care and ward facilities was critical in order to identify existing resources and needs. Based on the second questionnaire results, it was possible to assess the needs for quality cancer surgery. We realised the current organisational level of cancer care, all human resources of MCH will be fundamental in the organisation of oncology but most of them, including de surgeons, need specific training. We agree that is also important and crucial to creating a training program in oncology at the undergraduate level. The challenge is how we can do it effectively, taking into account local realities.

The complex nature of cancer makes oncological surgeries highly technically demanding and, therefore, outcomes are improved when surgery is undertaken by experienced multidisciplinary teams of specialists at high-workload centres and with adequate resources [13].

Our results show that surgeons participate erratically in the multidisciplinary therapeutic decision meeting. Multidisciplinary programs provide many benefits for the physicians involved [14, 15]. The attendance of multiple specialists from each discipline in this type of meetings leads to dynamic discussion and learning opportunities for all, especially students and trainees [16]. Multidisciplinary team management is associated with improved outcomes after surgery for breast, colorectal and oesophageal cancer [17, 18].

Stefan *et al* [19] address the various areas of oncology in a very interesting article on education and training for the future in cancer research at the East African Regional Meeting. Unfortunately, in their work, the training of surgical oncologists was not discussed [19]. However, the College of Surgeons of East, Central and Southern Africa developed a fellowship in surgical oncology and the average length of time it takes to train surgical oncologist ranges from 11 to 17–19 years [20]. However, this training is long and does not cover the immediate needs of Mozambique. It is necessary to combine a formal training with a fast-track program (2 years). Thus, in order to improve the quality of care in MCH, the creation of a comprehensive CEP to address the educational needs of medical students, interns, residents, fellows, nurses and allied health staff is crucial. However, cultural and university education differences of the Lusophone African countries must be taken into account. It is important that training in oncology and surgical oncology considers these differences.

Despite different interventions to train Mozambique oncologists involving Brazilian hospitals, the Calouste Gulbenkian foundation from Portugal, MD Anderson Cancer Center from USA and others, according to the respondents, there were no substantial changes in the practice of surgical oncology at MCH. It seems clear that without integrating these training efforts into an official and formal CEP at different levels (namely, pre-graduate and continuing medical education), the results will continue to be fragile and these activities will be erratic [21, 22].

A surgical oncologist should possess in-depth knowledge of malignancies involving each specific disease site. Thus, our proposal takes into account local resources, the nosological profile, the critical actors on the ground and the need to first create a group of competent trainers who later disseminate oncology training in general and oncology surgery in particular.

We follow the recommendations of Are *et al* [12], regarding the training and certification of surgical oncologists, namely: *The training period should be shortened, if possible. The training requirements can be tailored to suit the local needs based on the most prevalent malignancies diagnosed in the region. LMIACs can proactively partner with foreign countries that offer surgical oncology fellowships. Emphasis also needs to be placed on continuing medical education to remain abreast of the current standards of treatment. Training should include the basic principles of chemotherapy and other disciplines of oncology. Surgeons should also be equipped with knowledge on pain, palliative care in order to improve the quality of life for cancer patients* [20].

In the definition of the contents suggested in our programme, we adopted the global curriculum in surgical oncology suggested by Are *et al* [12], the recommendations of Mozambican surgeons, but we prioritise the surgical treatment of the most prevalent malignancies in Mozambique, as had already been done in other Lusophone African countries with success [23].

As a whole, during the 2 years surgical oncology fellowship programme (during surgical residency), the clinical rotation includes: 12 months spent in the surgical oncology department of a high-volume centre, focusing on complex oesophageal and gastrointestinal malignancies, pancreatic and hepatobiliary malignancies, breast cancer, melanoma, head and neck cancer, bone and soft-tissue sarcoma and foregut malignancies; 1 month in a radiation therapy unit; 1 month in a pathology unit and 1 month in a medical oncology department. The remaining 9 months will be spent in Mozambique conducting oncological research activity.

For formal general and thoracic surgeons, MCH should offer advanced training opportunities in surgical oncology (3 months) who have before completed the basic oncology training program and are interested in an additional intensive experience in advanced surgical oncology related to their specific area, namely, breast cancer, colorectal or oesophageal cancer. In our view, this methodology may apply to other countries with similar realities, daily activity and difficulties encountered should become teaching and research issues in order to overcome them properly. Specific topics, such as assessment based on decision making on assignment as trusted professional activities, is probably a useful methodological option in this context [24].

Thus, the proposal of our study should be discussed by the country’s health and university authorities and by the college of surgeons in order to integrate this Mozambican program for training surgical oncologists and then evaluate its effectiveness.

Similar programs should be implemented in other African countries, demonstrating their capacity to adapt to their specific reality and conditions. Those responsible for the training of surgical oncologists from countries with high resources should, according to the difficulties we have studied, develop specific and adapted courses that can be used as a complement of the training capacities of developing countries. For surgical oncology fellows in higher-resource settings specifically, the learning benefits of rotations in LMICs are even greater since they learn to deal with cancer patients at advanced stages or different cancer biology, in countries having fewer screening options, fewer imaging options, as well as limited perioperative care [25].

## Conclusions

The findings of this study contributed to the development of an educational program in surgical oncology considered essential for the training of surgeons and residents of surgery at MCH. Undergraduate medical training programmes should incorporate oncology education.

Cancer education should fully integrate all healthcare professionals involved in cancer care. The educational cancer program and the mobilisation of adequate resources will ensure the provision of adequate surgical oncology treatments at MCH. The training requirements should be tailored to suit the local needs based on the most prevalent malignancies diagnosed in the region. In our view, this methodology may apply to other countries with similar realities in the training of surgical oncologists.

## Conflicts of interest

Ellen Baker is the director of Project ECHO, MD Anderson, USA.

For the other authors, there are no conflicts of interest to disclose and no financial or commercial relationships.

## Authors’ contributions

This study was conceptualised, designed and written by Atílio Morais and Lúcio Lara Santos. Acquisition of data was carried out by Atílio Morais, Adriano Tivane, Matchecane Cossa, Jotamo Come, Volodimir Venetsky, Fernando Torres, Victor Pacheco, Miguel Reyes, Germano Pires. Analysis and interpretation of data were done by Atílio Morais, Mariana Peyroteo and Lúcio Lara Santos. Ellen Baker, Satish Tulsidas, Maria Rosário Martins, Moshin Sidat and Lúcio Lara Santos revised the article for important intellectual content. All authors read and agreed to the final version of this manuscript. Atílio Morais and Lúcio Lara Santos equally contributed to this study.

## Funding

This investigation had no financial support.

## Figures and Tables

**Table 1.** Specific resources lacking to perform breast, colorectal and esophageal cancer surgical treatment.

	Breast cancer	Colorectal cancer	Oesophageal cancer
**Diagnostic**	imaging resources and to perform core needle biopsy of the breast	Rigid sigmoidoscope	Imaging resources
**Surgery**	Patent Blue V for sentinel lymph node biopsy	Bookwalter retractor system	Surgical Clips and Clamps
	Fluorescence navigation with indocyanine green for detecting sentinel lymph nodes	Saint Mark’s pelvic retractor	Circular and linear staplers
	Surgical clips	Surgical stapler for rectum	Harmonic scalpel consumables
**Post-operative care**	Aspiration drainage	Provide information and advice on diet to stoma patients	Oesophageal stents

**Table 2.** Training capacity of Mozambique in surgery, anaesthetist and nurses.

Health professional	Academic level (degree)	Years of duration (academic degree, training + residency)	Rotation in oncology during studies	Number of certifying universities/institutions	Number graduates per year	Educational institutions*	
						Public sector	Private sector
Nurse	BSc	3 years	No	4	60	4	4
Nurse Anaesthetist	BSc, AN	2 + 3 years	No	1	10	1	
General practitioner	MD	6 years	Yes	8	200	4	4
Anaesthetist	MD + R	6 + 5 years	Yes	1	3	1	
Gynaecologist	MD + R	6 + 5 years	Yes	1	5	1	
Orthopaedist	MD + R	6 + 4 years	No	1	3	1	
Urologist	MD + R	6 + 5 years	Yes	1	1	1	
General surgeon	MD + R	6 + 5 years	Yes	1	4	1	
Thoracic surgeon	MD + R	6 + 5 years	Yes	1	1	1	
Plastic Surgeon	MD + R	6 + 5 years	Yes	1	1	1	

BSc = Bachelor of Science in Nursing; AN = Anaesthetic Nursing. MD = Medical Doctor graduation, R = Residency

* According to the Mozambican catalogue of higher education (www.mzformativa.com)

**Table 3.** MCH CEP (Modules).

Undergraduate level	General surgery residents	General surgeons*
Basics of oncology	Basics of oncology	
	Breast cancer	Breast cancer
	Colorectal cancer	Colorectal cancer
	Oesophageal cancer	Oesophageal cancer
	Surgical oncology fellowships	Advanced surgical oncology training

* According their oncological field and surgical expertise

**Table 4.** Modules and core content of the MCH CEP.

**Module 1—Basics of oncology**
Dimensions of the cancer problem in Mozambique; cancer registry; Carcinogenesis; The diagnosis in oncology; cancer prevention, cancer staging; surgical oncology; chemotherapy; radiotherapy; personalised treatment in oncology; multidisciplinary therapeutic approach for cancer; follow-up of the cancer patient; best supportive care; research in oncology; quality in oncological care
**Module 2—Breast cancer**
Breast cancer epidemiology; breast imaging and diagnostics, including screening; The molecular basis of breast cancer and pathological phenotypes; breast cancer classification; management of BRCA (breast cancer) gene carriers; treatment of ductal carcinoma in situ (DCIS); surgery: standards of care; sentinel node biopsy: technical aspects, intraoperative nodal assessment and localisation techniques; axillary lymph node dissection: indications and technical aspects; oncoplastic breast surgery; reconstructive surgery; Radiation therapy: standard of care; therapies in HR+ breast cancer; therapies in HER2+ breast cancer; therapies in triple negative breast cancer; management of advanced disease. Follow-up
**Module 3—Colorectal cancer**
Incidence and epidemiology; symptoms; diagnosis; pathology and molecular biology (RAS and BRAF mutational status); histopathology; staging and risk assessment; management of local and locoregional disease; colon and rectal surgery; laparoscopy approach; neoadjuvant and adjuvant treatment; Selection between short-course preoperative radiotherapy and long-course chemoradiotherapy; management of advanced/metastatic disease; treatment of liver and lung metastasis; management of local recurrence; follow-up
**Module 4—Oesophageal cancer**
Incidence; symptoms; pathogenesis; histological classification; diagnosis and staging; treatment of premalignant lesions with endoscopic therapy or esophagectomy; selection of appropriate treatment; pre-habilitation program; the selection of surgical approach; radiation treatment; combined chemoradiation; postoperative adjuvant chemotherapy or chemoradiation; endoscopic palliative therapy; chemotherapy for metastatic disease; nutritional support; follow-up
